# Selective Inhibition of Histone Deacetylases 1/2/6 in Combination with Gemcitabine: A Promising Combination for Pancreatic Cancer Therapy

**DOI:** 10.3390/cancers11091327

**Published:** 2019-09-07

**Authors:** Richard S. Laschanzky, Lisa E. Humphrey, Jihyun Ma, Lynette M. Smith, Thomas J. Enke, Surendra K. Shukla, Aneesha Dasgupta, Pankaj K. Singh, Gillian M. Howell, Michael G. Brattain, Quan P. Ly, Adrian R. Black, Jennifer D. Black

**Affiliations:** 1 Eppley Institute for Research in Cancer and Allied Diseases, University of Nebraska Medical Center, Omaha, NE 68198, USA (R.S.L.) (L.E.H.) (T.J.E.) (S.K.S.) (A.D.) (P.K.S.) (G.M.H.) (A.R.B.); 2 Department of Biostatistics, University of Nebraska Medical Center, Omaha, NE 68198, USA (J.M.) (L.M.S.); 3 Department of Surgery, Division of Surgical Oncology, University of Nebraska Medical Center, Omaha, NE 68198, USA

**Keywords:** pancreatic ductal adenocarcinoma, histone deacetylase, HDAC, histone deacetylase inhibitor, gemcitabine

## Abstract

Pancreatic ductal adenocarcinoma (PDAC) has a five-year survival rate of <10% due in part to a lack of effective therapies. Pan-histone deacetylase (HDAC) inhibitors have shown preclinical efficacy against PDAC but have failed in the clinic due to toxicity. Selective HDAC inhibitors may reduce toxicity while retaining therapeutic efficacy. However, their use requires identification of the specific HDACs that mediate the therapeutic effects of HDAC inhibitors in PDAC. We determined that the HDAC1/2/3 inhibitor Mocetinostat synergizes with the HDAC4/5/6 inhibitor LMK-235 in a panel of PDAC cell lines. Furthermore, while neither drug alone synergizes with gemcitabine, the combination of Mocetinostat, LMK-235, and gemcitabine showed strong synergy. Using small interfering (si)RNA-mediated knockdown, this synergy was attributed to inhibition of HDACs 1, 2, and 6. Pharmacological inhibition of HDACs 1 and 2 with Romidepsin and HDAC6 with ACY-1215 also potently synergized with gemcitabine in a panel of PDAC cell lines, and this drug combination potentiated the antitumor effects of gemcitabine against PDAC xenografts in vivo. Collectively, our data show that inhibition of multiple HDACs is required for therapeutic effects of HDAC inhibitors and support the development of novel strategies to inhibit HDACs 1, 2, and 6 for PDAC therapy.

## 1. Introduction

Pancreatic ductal adenocarcinoma (PDAC), the third leading cause of cancer death in the United States, is one of the most deadly forms of cancer, with a five year survival rate of less than 10% and a median survival following diagnosis of 6–12 months [1,2,3]. This dismal prognosis can be attributed to late presentation at diagnosis, with up to 80% of PDAC patients presenting with advanced disease for which there is no effective treatment [4,5]. Even early stage patients who are eligible for resection have a five-year survival of ≤31%, with a median overall survival of ≤34 months [6]. For late stage patients, and the majority of patients who progress after resection, chemotherapy is the only option; however, current therapies offer limited benefit. Until recently, gemcitabine was the main first line treatment, giving a median overall survival of 6–7 months [7,8]. The introduction of new standard-of-care combined chemotherapy regimens, FOLFIRINOX and nab-paclitaxel (Abraxane) plus gemcitabine, has modestly improved survival times, but median overall survival remains less than 12 months [7,8,9]. Because this increased survival comes with attendant treatment-emergent toxicities, only patients with high performance status are eligible for these combined therapies, and gemcitabine remains the mainstay treatment for patients with comorbidities or low performance status [8,10]. Thus, although recent advances have led to an increase in survival times for PDAC patients, these advances have been modest at best, and there is an urgent need for novel therapies for PDAC.

Histone deacetylase (HDAC) inhibitors are novel drugs that have attracted considerable attention for the treatment of cancer [11]. The HDAC family comprises 18 non-redundant lysine deacetylases that are divided into four broad classes based on their homology to yeast prototypes [12,13]. These classes fall into two groups based on their cofactor requirements: classical Zn^2+^-dependent HDACs 1–11 (classes I, IIa, IIb, and IV) and the NAD^+^-dependent sirtuins (Class III) [11,13]. HDACs oppose the action of protein lysine acetyltransferases and catalyze the deacetylation of lysines on multiple cellular proteins in addition to histones [11,13]. As such, they regulate many cellular processes that are important in tumorigenesis, including gene transcription, cell cycle progression, cell survival, DNA repair, protein trafficking and degradation, and cell migration [14]. Expression of HDACs can be ubiquitous or cell type-specific depending on the HDAC [15]. Intracellular localization of HDAC proteins is also highly class-specific, and the HDACs have both unique and overlapping functions [14,16].

Consistent with the biological roles of HDACs, HDAC inhibitors have shown preclinical efficacy against a broad spectrum of cancer types [17]. They have also shown considerable promise in the clinic. As single agents, HDAC inhibitors have demonstrated the greatest efficacy against hematological malignancies, with Vorinostat, Romidepsin, Belinostat, and Panobinstat approved for treatment of T-cell lymphomas and/or multiple myeloma (Mocetinostat has also been given orphan drug status for diffuse large B-cell lymphoma) [18]. In contrast, clinical trials of HDAC inhibitors as single agents have given disappointing results in solid tumors [18]. Thus, a major effort for the clinical application of these agents is to use them in combination with other chemotherapeutic agents [18].

As with other tumor types, preclinical studies have pointed to the potential of HDAC inhibitors in the treatment of PDAC. Multiple HDAC inhibitors have antiproliferative and cytotoxic activity as single agents and synergize with clinically relevant therapeutics (e.g., gemcitabine, irinotecan, decitabine, proteasome inhibitors, and radiation) to induce apoptosis in PDAC cell lines, both in vitro and as xenografts in mice [19,20,21,22,23]. However, the preclinical promise of these agents has not been seen in clinical trials to date. ClinicalTrials.gov lists over 20 clinical trials that involve HDAC inhibitors in PDAC; however, only phase I trials have been completed, with phase II trials either still ongoing or terminated, and no trials have progressed to phase III or IV. A likely explanation for the lack of success in clinical development of HDAC inhibitor-based therapies for PDAC is their toxicity. A common limitation of clinical trials with reported findings (mainly hematological studies) is dose limiting toxicity, which has necessitated dose reductions or changes in dose scheduling [24]. Thus, realization of the potential of HDAC inhibitors for therapy of solid tumors requires strategies that reduce their systemic toxicity.

Historically, the HDAC inhibitors used in clinical trials have been broad-spectrum pan-HDAC inhibitors, such as Panobinostat, Vorinostat, and Belinostat. More recently, considerable effort has been dedicated to the development of more selective inhibitors of HDACs with the goal of retaining the antitumor effects of the pan-HDAC inhibitors while reducing toxicity associated with broad inhibition of multiple HDAC family members [25]. However, successful development of this strategy requires knowledge of the specific HDACs whose inhibition mediates antitumor effects as well as identification of HDACs that are dispensable as targets. To this end, the current study used selective HDAC inhibitors and siRNA-mediated knockdown to identify HDACs 1, 2, and 6 as key targets in the cytotoxic effects of pan-HDAC inhibition in a panel of PDAC cell lines. Findings from this work further demonstrate that selective inhibition of the activity of these HDACs cooperates with the clinically relevant chemotherapeutic, gemcitabine, in reducing PDAC cell viability in vitro and the growth of PDAC xenografts in vivo.

## 2. Results

### 2.1. Inhibition of Multiple HDACs Is Required for the Effects of HDAC Inhibitors on PDAC Cells

**Figure 1:**
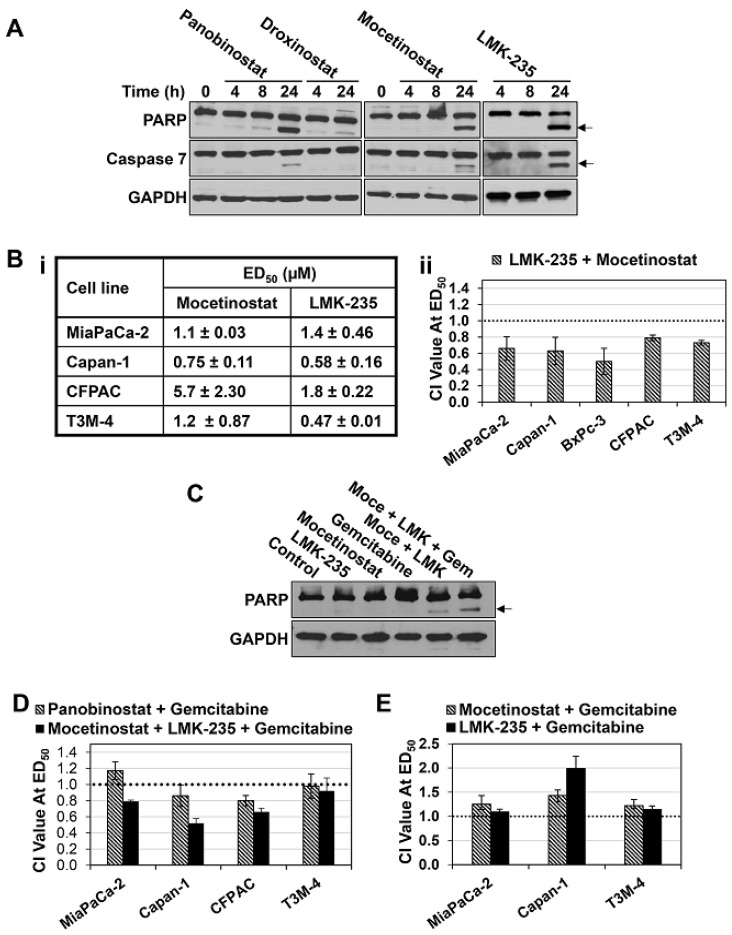
Synergistic interactions of Mocetinostat, LMK-235, and gemcitabine in pancreatic ductal adenocarcinoma (PDAC) cells. (**A**) MiaPaCa-2 cells treated with 100 nM Panobinostat, 10 µM Droxinostat, 10 µM Mocetinostat, or 10 µM LMK-235 for the indicated times and analyzed for poly (ADP-ribose) polymerase (PARP), caspase 7, and GAPDH expression by Western blotting. Arrow indicates the migration of cleaved proteins. (**B.i**) ED_50_ values for Mocetinostat and LMK-235 in pancreatic cancer cell lines. (**B.ii**) The indicated cell lines were treated with LMK-235, Mocetinostat, or a combination of LMK-235 and Mocetinostat at a constant ratio. After 72 h, 3-(4,5 Dimethylthiazol-2-yl)-2,5-diphenyltetrazoliumbromide (MTT) assays were performed, and the combination index (CI) value for the combination treatment is presented. Dotted line indicates CI value of one, which divides synergistic interaction (CI < 1) from antagonistic interaction (CI > 1). (**C**) Capan-1 cells were treated with LMK-235 (LMK, 600 nM), Mocetinostat (Moce, 1 µM), and/or gemcitabine (Gem, 80 nM) for 24 h, and expression of the indicated proteins was analyzed by Western blotting. Arrow indicates the migration of cleaved PARP. (**D**,**E**) As in **B** except that cells were treated with the indicated drugs either alone or in combination at equal ratios. Data are representative (**A**,**C**) or means ± s.e. (**B**,**D**,**E**) of at least three independent experiments.

To assess the potential of using selective HDAC inhibitors to circumvent the toxicity seen with pan-HDAC inhibitors, we tested the effects of more selective inhibitors on PDAC cells. The compounds evaluated included Mocetinostat, which is selective for HDACs 1, 2, and 3 [26], LMK-235, which targets HDACs 4, 5, and 6 [27], and Droxinostat, which is selective for HDACs 3, 6, and 8 [28]. The effects of these agents in PDAC cells were compared with those of the potent pan-HDAC inhibitor, Panobinostat, which targets all eleven classical HDACs in the nanomolar range [29]. At a 10 µM concentration, Droxinostat showed weak apoptosis-inducing activity in MiaPaCa-2 pancreatic cancer cells, reflected in weak to undetectable induction of poly (ADP-ribose) polymerase (PARP) and caspase 7 cleavage (Figure 1A, arrows). In contrast, as seen with Panobinostat, both Mocetinostat and LMK-235 robustly induced apoptosis in these cells, as confirmed by the accumulation of cleaved PARP and caspase 7 (Figure 1A, arrows). Consistent with their ability to induce apoptosis, Mocetinostat and LMK-235 reduced the viability of a panel of PDAC cell lines, as assessed by 3-(4,5 Dimethylthiazol-2-yl)-2,5-diphenyltetrazoliumbromide (MTT) assays following 72 h of treatment, with ED_50_ values ranging from 0.75 to 5.7 µM and 0.47 to 1.8 µM, respectively (Figure 1Bi). These data pointed to HDACs 1, 2, and/or 3, and HDACs 4, 5, and/or 6 in the cytotoxic effects of Panobinostat in PDAC cells.

To further explore these findings, the effects of combinations of Mocetinostat and LMK-235 on PDAC cell viability were examined using MTT assays, and the data were analyzed using the Chou–Talalay method [30,31]. This analysis provides a combination index (CI) value that indicates if the interaction is synergistic (CI < 1), additive (CI = 1), or antagonistic (CI > 1). Treatment of a panel of pancreatic cancer cell lines (MiaPaCa-2, Capan-1, BxPC-3, CFPAC, and T3M-4) with Mocetinostat and LMK-235 in combination resulted in CI values ranging from 0.5–0.8 for effects on cell viability (Figure 1Bii), pointing to a synergistic interaction between these compounds. This effect was supported by the ability of the drugs to cooperatively induce apoptosis in PDAC cells; while a 24 h treatment with 0.6 µM LMK-235 or 1 µM Mocetinostat had minimal effects on PARP cleavage in Capan-1 cells, when these drugs were combined, cleavage of PARP could be readily detected (Figure 1C, arrow). Thus, the potency of pan-HDAC inhibitors appears to result from inhibition of multiple HDACs with at least one being a class I HDAC (HDAC1, 2, or 3) and one being a class II HDAC (HDAC 4, 5, or 6).

The requirement for combined inhibition of one or more class I and class II HDACs was supported by analysis of the effects of HDAC inhibitors in combination with gemcitabine. As demonstrated by others [32], Panobinostat showed additive to mildly synergistic effects in combination with gemcitabine in PDAC cells (Figure 1D). While a combination of gemcitabine with either Mocetinostat or LMK-235 at ED_50_ was additive to antagonistic in these cells (Figure 1E), Mocetinostat and LMK-235 in combination synergized with gemcitabine in all cell lines tested except T3M-4, where the effect was additive (Figure 1D). Notably, this effect was more robust than that of the Panobinostat/gemcitabine combination in most PDAC cell lines (Figure 1D). As with the effects of the Mocetinostat/LMK-235 combination, this synergism can be attributed, at least in part, to enhanced apoptosis (Figure 1C). While 80 nM gemcitabine alone had little effect on PARP cleavage in Capan-1 cells after 24 h of treatment, addition of this concentration of gemcitabine potentiated PARP cleavage induced by the Mocetinostat (1 µM)/LMK235 (0.6 µM) combination (Figure 1C, arrow).

### 2.2. Inhibition of HDACs 1, 2, and 6 Accounts for the Effects of HDAC Inhibitors in PDAC Cells

**Figure 2:**
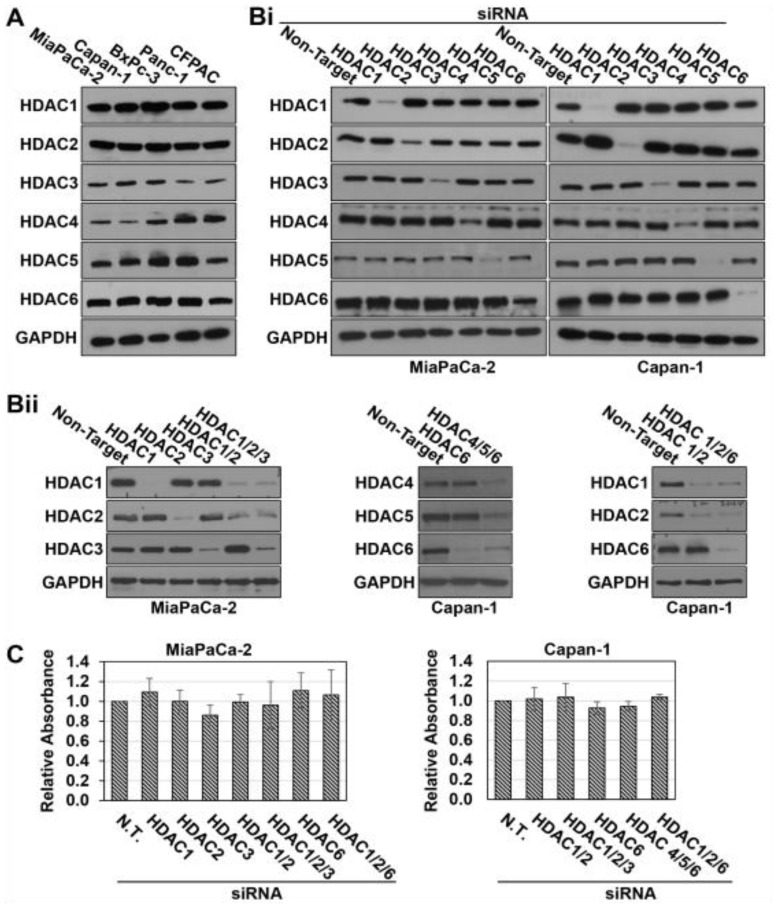
Knockdown of pan-histone deacetylase (HDACs) 1–6 in PDAC cells. (**A**,**B**) Western blot analysis of HDAC1, 2, 3, 4, 5, and 6 expression in the indicated cell lines. Cells were either untransfected (**A**) or transfected with non-targeting small interfering (si)RNA or ONTARGET*plus* SMARTPool siRNAs (each including four different siRNAs) targeting the indicated HDACs 48 h prior to harvest (**Bi**,**ii**). (**C**) MiaPaCa-2 and Capan-1 cells were transfected with the indicated siRNAs, and cell viability was determined by MTT assay 48 h after transfection. Data are representative (**A**,**B**) or means ± s.e. (**C**) of at least three independent experiments.

To identify the specific HDACs contributing to the synergism between Mocetinostat and LMK-235, we surveyed HDAC expression in PDAC cell lines. HDACs 1–6, the targets of the HDAC inhibitor combination, were expressed in all of the five PDAC cell lines tested, with HDAC3 and HDAC4 showing the most variability in expression between the lines (Figure 2A). The involvement of individual HDACs was further explored in MiaPaCa-2 and Capan-1 cells using siRNA technology. HDACs were knocked down individually or in combination using ON-TARGET plus SMARTpool siRNAs, which consist of a pool of four siRNAs designed and modified to increase specificity and reduce off-target effects. As shown in Figure 2Bi,ii, HDACs 1–6 could be effectively knocked down in Capan-1 cells, both singly and in combination, without affecting expression of other HDACs. Similar results were seen in MiaPaCa-2 cells, except that efficient knockdown of HDAC6 was difficult to achieve in this cell line (Figure 2Bi,ii).

**Figure 3:**
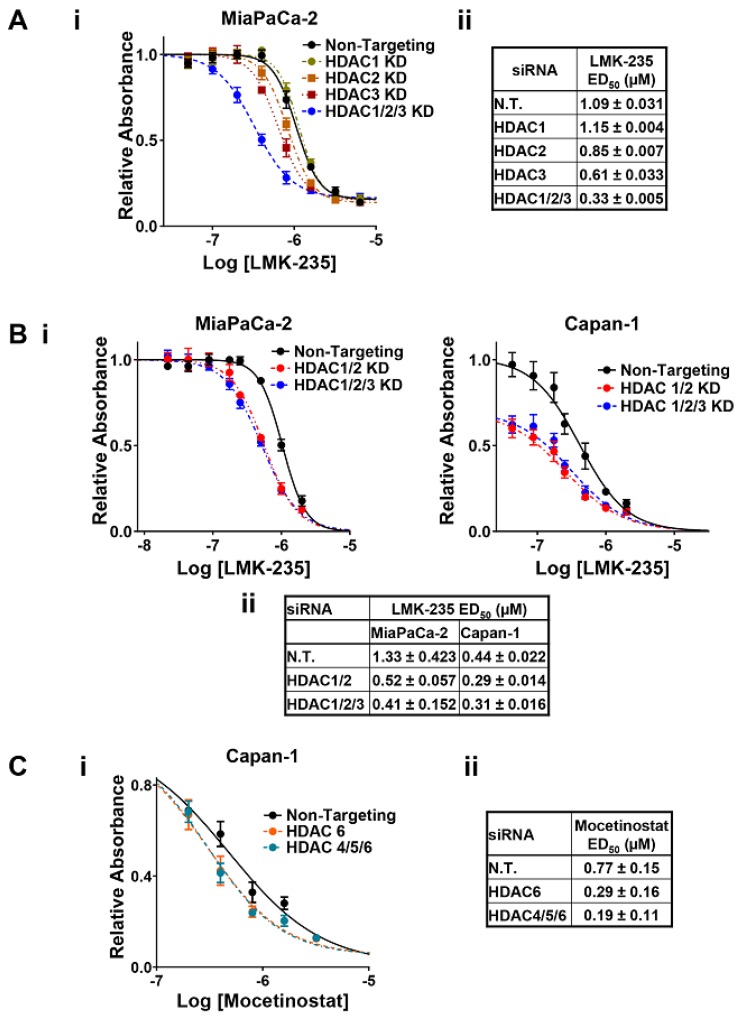
HDACs 1, 2, and 6 account for the synergism between Mocetinostat and LMK-235 in PDAC cells. HDACs 1, 2, and 3 (**A**,**B**) or HDACs 4, 5, and 6 (**C**) were knocked down (KD) in MiaPaCa-2 and/or Capan-1 cells by transfection with ONTARGET*plus* SMARTPool siRNAs targeting specific HDACs either individually or in combination, as indicated. Control cells were treated with non-targeting (N.T.) siRNA and, 24 h after transfection, cells were treated with various concentrations of LMK-235 (**A**,**B**) or Mocetinostat (**C**) and subjected to MTT assay after 72 h of drug treatment. (**i**) Representative graph of the effect of various concentrations of LMK-235 or Mocetinostat on the relative number of viable cells (absorption at 570 nm) following knockdown of the indicated HDACs (mean ± s.d. of six technical replicates for each condition). (**ii**) ED_50_ values for LMK-235 (**A**,**B**) or Mocetinostat (**C**) in cells transfected with the indicated siRNAs (mean ± s.d. for three biological replicates).

Knockdown of HDACs 1, 2, and 3 (the targets of Mocetinostat) or HDACs 4, 5, and 6 (the targets of LMK-235) was not toxic to the cells (Figure 2C). These data indicate that (a) a higher level of inhibition than obtainable with knockdown is needed for the toxicity seen with pharmacological inhibition of these HDACs, or (b) Mocetinostat and LMK-235 have off-target effects on the viability of PDAC cells that are independent of their effects on HDACs. Nonetheless, several lines of evidence point to an important role of HDAC inhibition in the synergy between the two drugs. As shown in Figure 3, combined knockdown of HDACs 1, 2, and 3 potentiated the cytotoxic effects of LMK-235 in both MiaPaCa-2 and Capan-1 cells, as indicated by a leftward shift in its dose response curves and reduced ED_50_ values (Figure 3A,B, *p* < 0.05). Notably, while individual knockdown of these HDACs was less effective in enhancing the response of cells to LMK-235 (Figure 3A), combined knockdown of HDACs 1 and 2 was as effective as knocking down all three HDACs (Figure 3B). Thus, inhibition of HDAC1 and 2 appears to be sufficient to mediate the synergistic effects of Mocetinostat with LMK235. In the converse experiments, knockdown of HDACs 4, 5, and 6 similarly reduced the ED_50_ of Mocetinostat in Capan-1 cells (Figure 3C, *p* = 0.01). Strikingly, the effects of knocking down HDAC6 alone on the ED_50_ of Mocetinostat (*p* = 0.01) recapitulated the effects of combined knockdown of HDACs 4, 5, and 6 (Figure 3C), indicating that inhibition of HDAC6 mediates the synergy between LMK-235 with Mocetinostat. Similar effects were not observed in MiaPaCa-2 cells (Laschanzky, R.S. and Black, J.D., unpublished results), presumably reflecting the less effective knockdown of HDAC6 achieved in these cells (see Figure 2B). Collectively, these data demonstrate that (a) the synergy between Mocetinostat and LMK-235 can be attributed, at least in part, to their effects on HDACs, and (b) this synergy is primarily due to the ability of the drugs to inhibit HDACs 1, 2, and 6.

### 2.3. Pharmacological Inhibition of HDACs 1, 2, and 6 Synergizes with Gemcitabine in PDAC Cells

**Figure 4:**
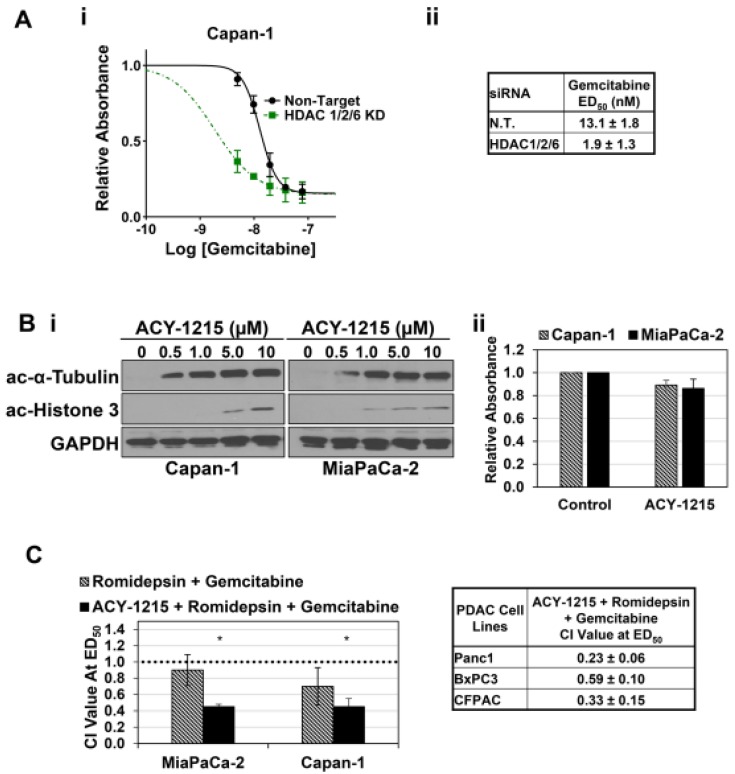
Inhibition of HDACs 1, 2, and 6 cooperates with gemcitabine. (**A.i,ii**) Capan-1 cells were transfected with non-targeting siRNA (N.T.) or ONTARGET*plus* SMARTPool siRNAs targeting HDACs 1, 2, and 6 and treated with gemcitabine as indicated for 72 h. (**B.i**) Capan-1 and MiaPaCa-2 cells were treated with the vehicle or the indicated concentrations of ACY-1215 for 24 h, and expression of acetyl-α-Tubulin (ac-α-Tubulin), acetyl-Histone 3 (ac-Histone 3), and GAPDH was assessed by Western blotting. (**ii**) The indicated cells were treated with 1 µM ACY-1215, and the number of viable cells was determined after 72 h. (**C**) The indicated cell lines were treated with Romidepsin, gemcitabine, or a combination of Romidepsin and gemcitabine at a constant ratio in the presence or the absence of 1 µM ACY-1215. After 72 h, CI values for the combination treatment were calculated as in Figure 1. * Significantly different from Romedepsin + Gemcitabine, *p* < 0.03. Data are representative or means ± s.e. of at least three independent experiments.

Having identified HDACs 1, 2, and 6 as relevant targets in the synergistic effects of Mocetinostat and LMK-235 in PDAC cells, we tested the effects of HDAC1, 2, and 6 knockdown on the cytotoxicity of gemcitabine, which continues to be a mainstay in the treatment of pancreatic cancer [8,10]. As seen with other HDAC knockdown combinations, simultaneous knockdown of HDACs 1, 2, and 6 was not toxic to the cells (Figure 2C); however, as expected from the synergy between Mocetinostat, LMK235, and gemcitabine, knockdown of HDACs 1, 2, and 6 led to a substantial increase in the cytotoxicity of gemcitabine, resulting in a ~7-fold reduction in its ED_50_ (Figure 4A, *p* < 0.01).

To further explore the therapeutic potential of the interaction between selective HDAC1, 2, and 6 inhibition and chemotherapeutic agents in PDAC, we evaluated the synergy between gemcitabine and combinations of the Food and Drug Administration (FDA)-approved HDAC1/2 selective inhibitor, Romidepsin [33,34], and the HDAC6 selective inhibitor, ACY-1215 [34]. HDAC6 selective inhibitors, including ACY-1215, have been reported to be non-toxic in other systems [35,36]. Therefore, the effects of ACY-1215 on acetylation of α-tubulin, an established substrate of HDAC6 [37], were assessed by Western blotting to determine an appropriate dose of the drug. Maximal effects on α-tubulin acetylation were achieved with doses between 0.5 and 1 µM of ACY-1215 (Figure 4Bi); thus, ACY-1215 was used at 1 µM in future experiments. This dose of ACY-1215 had only a mild effect on the viability of Capan-1 and MiaPaCa-2 cells (Figure 4Bii); however, higher doses were toxic to these cells (Laschanzky, R.S. and Black, J.D., unpublished results), presumably reflecting off-target effects.

Since ACY-1215 is not cytotoxic at concentrations that inhibit HDAC6 activity, the mass action formulas underlying the Chou–Talalay method could not be directly used to examine the interaction between pharmacological inhibition of HDACs 1, 2, and 6 and gemcitabine in reducing PDAC cell viability. Therefore, the effects of ACY-1215 on the interaction between Romidepsin and gemcitabine were evaluated by treating PDAC cells with varying concentrations of Romidepsin and gemcitabine at a constant ratio in the presence or the absence of 1 µM ACY-1215. As seen with the HDAC1/2/3 inhibitor Mocetinostat, Romidepsin had an additive to mildly synergistic effect on cell viability when combined with gemcitabine in MiaPaCa-2 and Capan-1 cells (Figure 4C). However, when ACY-1215 was included, a marked increase in synergism was observed (Figure 4C), consistent with the synergism seen with combinations of Mocetinostat, LMK-235, and gemcitabine (Figure 1D). Thus, consistent with the effects of siRNA-mediated knockdown, pharmacological inhibition of HDACs 1, 2, and 6 augments the cytotoxic effects of gemcitabine in PDAC cells. Furthermore, these data confirm a role for HDAC6 in synergistic interactions with gemcitabine in MiaPaCa-2 cells, which were not amenable to analysis in the knockdown experiments (see above). Similar CI values were noted for the combination in Panc1, BxPC3, and CFPAC cells (Figure 4C, right panel).

### 2.4. Selective Inhibition of HDACs 1, 2, and 6 Increases the Apoptosis-Inducing Effects of Gemcitabine In Vitro and Augments the Antitumor Effects of Gemcitabine In Vivo

**Figure 5:**
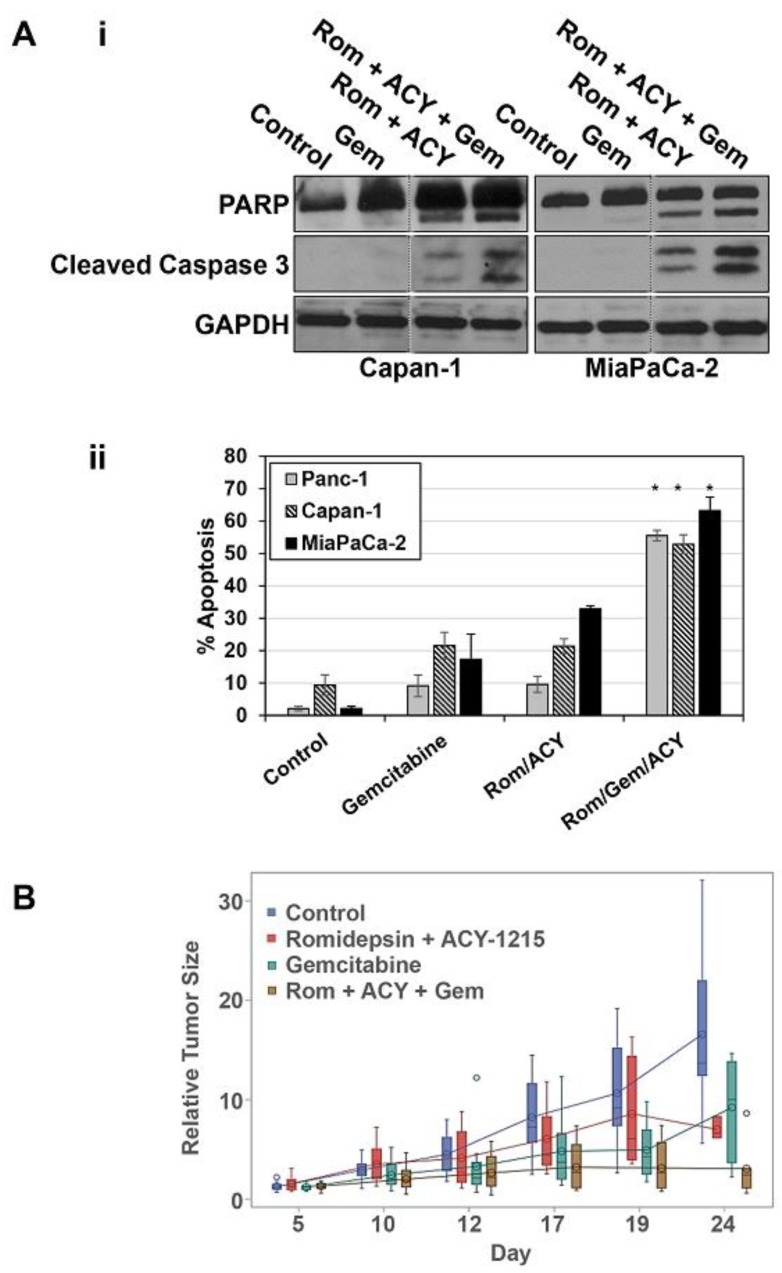
Pharmacological inhibition of HDACs 1, 2, and 6 enhances the effects of gemcitabine on PDAC cells in vitro and in vivo. (**A.i**) The indicated cells were treated with vehicle, 40 nM gemcitabine (Gem), 8 nM Romidepsin (Rom), and/or 1 µM ACY-1215 (ACY) for 48 h, and the expression of PARP, cleaved caspase 3, and GAPDH was determined by Western blotting. Vertical lines indicate rearrangement of lanes from a single membrane for clarity. Data are representative of three independent experiments. (**A.ii**) PDAC cells treated as in (**i**) were analyzed for apoptosis based on cell surface phosphatidylserine using Annexin V-FITC. Data are means ± s.e. * Significantly different from Gemcitabine and Rom/ACY, *p* < 0.05, n = 3. (**B**) Athymic nude mice bearing subcutaneous Capan-1 xenografts (16 per group) were given intraperitoneal injections of vehicle, Romidepsin plus ACY-1215, gemcitabine, or Romidepsin plus ACY-1215 plus gemcitabine, as described in Materials and Methods. Tumor volumes at various time points were normalized to the volume on day 0 for each mouse. Changes in tumor growth were analyzed with a mixed model and are displayed with 95% confidence intervals.

To determine the ability of selective HDAC 1, 2, and 6 inhibition to augment the therapeutic effects of gemcitabine in vivo, the impact of combined Romidepsin/ACY-1215 treatment on effects of gemcitabine was examined in mice bearing PDAC xenografts (UNMC IACUC approval code: 18-177-01-FC). The use of this combination in mice was further supported by in vitro analysis of the effects of Romidepsin, ACY-1215, and gemcitabine on apoptosis in PDAC cells. As shown in Figure 5A, addition of Romidepsin and ACY-1215 to gemcitabine markedly enhanced apoptosis in pancreatic cancer cells, as indicated by PARP and caspase 3 cleavage as well as Annexin V staining (Figure 5Ai,ii). Based on reported doses in mice of 2 mg/kg, twice weekly for Romidepsin and 30–50 mg/kg, five days per week for ACY-1215 (e.g., [38,39,40,41,42]), we tested a combination of 2 mg/kg Romidepsin and 40 mg/kg ACY-1215 in toxicity studies. Nude mice treated with 2 mg/kg Romidepsin and 40 mg/kg ACY-1215 showed similar weight gain to vehicle treated mice over a three-week period (22% ± 1.4% vs. 21% ± 1.1%), indicating that the combination was well tolerated; therefore, these doses were used in xenograft studies.

To model the use of gemcitabine in the clinic, mice were treated with gemcitabine at 640 mg/kg twice weekly, which is 80% of the maximum tolerated dose. The Romidepsin/ACY-1215 combination had a mild inhibitory effect on the growth of Capan-1 xenografts (Figure 5B). Although gemcitabine was initially able to inhibit tumor growth in this model, the effect was temporary, and tumor growth resumed as a resistant population of cells grew out. Notably, treatment with gemcitabine in combination with Romidepsin and ACY-1215 markedly inhibited this later growth of tumors, leading to a statistically significant reduction in tumor size by 24 days compared with gemcitabine treatment alone (Figure 5B). Thus, consistent with the in vitro data, selective HDAC1, 2, and 6 inhibitors are able to potentiate the effects of gemcitabine on PDAC tumors in vivo, blocking the ability of these tumors to grow following prolonged gemcitabine treatment.

## 3. Discussion

The dose limiting toxicities of pan-HDAC inhibitors in patients have led to the development of selective inhibitors, based on the concept that targeting a subset of HDACs would reduce toxicity while retaining the promising anti-tumor activity of these agents [25]. However, rational development of this strategy requires knowledge of the critical HDAC targets that underlie the therapeutic effects of HDAC inhibitors in a particular cancer type. Here, we used a variety of selective HDAC inhibitors coupled with siRNA-mediated knockdown of class I and class II HDACs in PDAC cells to determine that the effects of pan-HDAC inhibitors require inhibition of three HDACs from two different classes. Mocetinostat and LMK-235, inhibitors of class I and class II HDACs, respectively, had synergistic effects on PDAC cell viability. This effect could be attributed to HDAC inhibition since siRNA-mediated knockdown of class I or class II HDACs augmented the cytotoxic effects of LMK-235 and Mocetinostat, respectively. Further analysis revealed that this cooperativity could be accounted for by inhibition of HDACs 1, 2, and 6. Cooperativity between inhibition of class I HDACs and HDAC6 is consistent with the ability of Mocetinostat (MGCD0103) to synergize with the HDAC6 specific inhibitor, Tubastatin A, in BxPc3 and Panc1 PDAC cells in vitro [36]. However, to our knowledge, our study is the first to define the specific class I HDACs whose inhibition mediates this cooperativity. Furthermore, we demonstrate that inhibition of HDACs 1, 2, and 6 synergistically enhances the cytotoxic effects of the clinically relevant chemotherapeutic agent, gemcitabine, in vitro and potentiates its anti-tumor activity in vivo.

Our identification of HDACs 1, 2, and 6 as critical players in the therapeutic effects of HDAC inhibitors likely explains the lack of success of selective HDAC inhibitors in clinical trials for PDAC. The selective class I HDAC inhibitors CI-994 (Tacedinaline) and Mocetinostat have been tested in combination with gemcitabine in phase II trials for the treatment of patients with advanced PDAC; however, the combinations were found to offer no advantage compared with gemcitabine alone, and further trials were not recommended [43,44]. A phase I trial of Romidepsin in combination with gemcitabine for PDAC patients has also been completed [45], but this combination has not advanced to phase II. In light of our finding that the combination of Mocetinostat and gemcitabine is antagonistic in PDAC cells, these findings are not surprising. However, while the class II selective LMK-235 also showed antagonism with gemcitabine in these cells, the combination of Mocetinostat and LMK-235 with gemcitabine showed strong synergy. This synergy can be attributed, at least in part, to the combined inhibition of HDACs 1, 2, and 6 since (a) knockdown of these HDACs potentiated the effects of gemcitabine in PDAC cells and (b) a combination of the HDAC 1/2 inhibitor, Romidepsin, and the HDAC6 inhibitor, ACY-1215, also synergized with gemcitabine in vitro and augmented the effects of gemcitabine in vivo.

Although the molecular factors underlying the requirement for simultaneous inhibition of HDACs 1, 2, and 6 in PDAC cells are not known, several studies point to a possible basis for the therapeutic advantage of this combination. HDAC1 is overexpressed in 40% of PDACs and is associated with a significantly worse prognosis [46,47]. Similarly, HDAC2, a well-established tumor promoter, is also overexpressed in a majority of PDACs [47]. Given the reported ability of HDAC1 to compensate for HDAC2 loss [48], a need for simultaneous inhibition of these two enzymes is not unexpected. It is notable in regard to our findings that HDACs 1 and 2 downregulate E-cadherin in PDAC cells [49], and E-cadherin loss is associated with resistance to gemcitabine and other chemotherapeutics [50,51]. Interactions between HDAC1/2 and HDAC6 have also been observed in PDAC cells. HDAC2 and HDAC6 cooperate to promote the loss of primary cilia during PDAC tumorigenesis through a mechanism that involves HDAC2-mediated upregulation of Aurora A and subsequent increased phosphorylation/activation of HDAC6 [52]. Effects on p53 may also underlie the need for simultaneous inhibition of HDACs 1, 2, and 6. HDAC6 inhibition selectively destabilizes mutant p53 protein while promoting the acetylation and the activity of wild-type p53 [53,54]. In contrast, HDAC1/2 inhibition downregulates both mutant and wild-type p53 at the transcriptional level [55]. Thus, simultaneous inhibition of these three HDACs may be required for maximal restoration of p53 signaling in PDAC cells.

In contrast to the effects of many HDAC inhibitors, knockdown of HDACs 1–6 alone or in combination was not toxic to PDAC cells. Although this may reflect lower levels of HDAC inhibition than can be achieved with pharmacological agents, off-target effects of the HDAC inhibitors are also likely to contribute to the difference. This can be seen with ACY-1215—consistent with findings that HDAC6 inhibition is not inherently cytotoxic to PDAC cells [35,36], ACY-1215 only affected cell viability at concentrations higher than those needed for complete inhibition of HDAC6-mediated deacetylation of α-tubulin. Off-target effects may also be a factor for other HDAC inhibitors, including selective class I inhibitors such as Entinostat and Romidepsin [56,57]. Discrepancies between the cytotoxicity of these agents and their ability to affect histone acetylation have been noted, indicating that non-epigenetic off-target effects may partially underlie their anticancer effects. Importantly, our data show that there is a therapeutic window in which non-toxic levels of HDAC 1, 2, and 6 inhibition can potentiate the effects of chemotherapeutics. This finding points to the potential of (a) generating more specific selective HDAC inhibitors with reduced off-target effects and (b) developing treatment regimens using non-toxic levels of HDAC 1, 2, and 6 inhibitors to augment the effects of chemotherapeutic agents.

## 4. Materials and Methods

### 4.1. Cell Culture

Human PDAC cell lines, MiaPaCa-2 (ATCC CRL-1420), Capan-1 (ATCC HTB-79), BxPC-3 (ATCC CRL-1687), CFPAC-1 (ATCC CRL-1918), T3M-4 (Riken RCB1021), and PANC-1 (ATCC CRL-1469) were maintained in McCoy’s 5A medium supplemented with pyruvate, vitamins, amino acids, antibiotics, and 10% fetal bovine serum at 37 °C in 5% CO_2_. Where indicated, cells were treated with Panobinostat (Sellechem, Houston, TX, USA), Mocetinostat (Sellechem), LMK-235 (Sellechem), ACY-1215 (MedChem Express, Monmouth Junction, NJ, USA), Romidepsin (MedChem Express), and/or gemcitabine (Sagent Pharmaceuticals, Schaumburg, IL, USA) in DMSO (except gemcitabine, which was in sterile water). For controls, cells were treated with vehicle; DMSO concentrations in the final culture medium were ≤ 0.001%.

### 4.2. Western Blot Analysis

Cells were lysed in 50 mM Tris-HCl, pH 7.4, 100 mM NaCl, 1% Nonidet P-40, 2 mM EDTA, 0.1% SDS, 50 mM NaF, 10 mM Na3VO4, 1 mM phenylmethylsulfonyl fluoride, 25 μg/mL β-glycerophosphate, and one protease inhibitor mixture tablet from Roche, and extracts (30 μg protein) were analyzed by Western blotting as described [58]. Primary antibodies were from Cell Signaling [PARP: 95425; Caspase 7: 128275; GAPDH: 5174; HDAC1: 3565; HDAC2: 51135; HDAC3 39495; HDAC4: 7628; HDAC5: 204585; HDAC6: 76125; acetyl-α-tubulin (Lys40): 5335; acetyl-histone 3: 9649; cleaved caspase 3: 9664]. Following transfer, membranes were cut (guided by molecular weight markers, as detailed in Supplementary Material S2) to facilitate probing for multiple proteins at specific molecular weights. Densitometry of bands from scanned films was performed using Image J software (NIH), and the intensities relative to loading control are included in Supplementary Material S1.

### 4.3. MTT Assay

Cells were plated at 2 × 10^3^ (MiaPaCa-2) or 3 × 10^3^ (Capan-1) cells per well in 96-well plates and treated with the indicated concentrations of drug (or vehicle) after 24 h. Cell viability was assessed after 72 h of drug treatment using 3-(4,5 Dimethylthiazol-2-yl)-2,5-diphenyltetrazoliumbromide (MTT) as described previously [59].

### 4.4. siRNA Knockdown

Human ON-TARGET*plus* SMARTpool siRNAs against HDACs 1, 2, 3, 4, 5, and 6 and non-targeting siRNA were from Dharmacon. ON-TARGET*plus* SMARTPool siRNAs consist of four siRNAs that are designed and modified to increase specificity and reduce off-target effects. MiaPaCa-2 and Capan-1 human PDAC cells were transfected with 37.5 pmol each of targeted and non-targeting siRNA using Lipofectamine 3000 (Thermo Fisher Scientific, Waltham, MA, USA) according to the manufacturer’s protocol. After 48 h, cells were analyzed by Western blotting or subjected to drug treatment and MTT assay.

### 4.5. Annexin V-FITC assay

Cell lines were plated at 2.5 × 10^5^ cells in 10 cm culture plates and treated with HDAC inhibitors and/or gemcitabine after 24 h. Following 30 h of drug treatment, apoptosis (cell surface phosphatidylserine) was assessed using the Annexin V-FITC Apoptosis Detection Kit (Sigma, St. Louis, MO, USA) as prescribed by the manufacturer. Flow cytometry was performed in the Flow Cytometry Research Facility at the University of Nebraska Medical Center (UNMC).

### 4.6. Mouse Xenograft Studies

All animal experiments were performed under a protocol approved by the IACUC at UNMC (approval code: 18-177-01-FC). Capan-1 cells (5 × 10^6^) were injected into the flanks of athymic NCr-nu/nu mice (Charles River, Durham, NC, USA). After tumors had reached ~1000 mm^3^, 16 mice per group were treated with vehicle, 2 mg/kg Romidepsin plus 40 mg/kg ACY-1215, 640 mg/kg gemcitabine, or 2 mg/kg Romidepsin plus 40 mg/kg ACY-1215 plus 640 mg/kg gemcitabine for 3 weeks. Treatment schedules were as follows: 40 mg/kg ACY-1215, daily; 2 mg/kg Romidepsin, twice weekly; 640 mg/kg gemcitabine, twice weekly. Tumors were measured daily using calipers, and volumes were estimated using the formula 3.14/6 × length × width^2^.

### 4.7. Analysis of Drug Interactions

Analysis of drug combination effects and combination index (CI) was performed using the method of Chou and Talalay [30,31]. Cell lines were treated with drugs individually and in combination at equipotent ED_50_ ratios for each drug for 72 h, and CI values were calculated from the results of MTT assays using Calcusyn software (Biosoft, Cambridge, UK). CI > 1 indicates antagonism between drugs, CI = 1 indicates additive effects, CI < 1 indicates drug synergy.

### 4.8. Statistics

Curve fitting and estimation of ED_50_ values for drug treatments were performed using GraphPad Prism Software (GraphPad Software, San Diego, CA, USA). Statistical differences were determined by Student’s *t* test using Microsoft Excel (*p* < 0.05 was considered statistically significant). For xenograft growth, descriptive statistics and mean plot (counts, means, standard deviations, minimums, and maximums) were used to summarize the tumor growth for treatment days and groups. Measurements of each outcome observed over treatment days were considered as repeated measures and fit with a linear mixed effect model with AR(1) structure for correlations to evaluate the change of the outcome measurement values (tumor growth) between treatment groups. In addition, pairwise comparisons of outcome measurements in treatment groups were compared in each measurement time point and adjusted for multiple comparisons with Tukey’s method. Significance was determined by including interaction terms of treatment day and group in the mixed effect models. All analyses were done using SAS, Version 9.4 (SAS, Cary, NC, USA). *p* < 0.05 was considered statistically significant.

## 5. Conclusions

Using selective HDAC inhibitors and siRNA-mediated knockdown in PDAC cells, this study demonstrates that the therapeutic effects of pan-HDAC inhibitors result from combined repression of the activity of multiple HDACs from different classes. Therefore, development of treatments based on selective HDAC inhibitors requires detailed knowledge of the relevant HDACs in a particular cancer type. Our analysis determined that HDACs 1, 2, and 6 are major targets of pan-HDAC inhibitors in PDAC and that non-toxic levels of inhibition of selective HDACs can augment the effects of chemotherapeutic agents. Inhibition of HDACs 1, 2, and 6 cooperates with the anticancer effects of gemcitabine both in vitro and in vivo. Thus, our findings point to development of treatment regimens that target HDACs 1, 2, and 6 as a therapeutic option for management of PDAC.

## Supplementary Materials

The following are available online at https://www.mdpi.com/2072-6694/11/9/1327/s1, Supplementary Material S1: Densitometry analysis of Western blots (band intensity normalized to loading control), Supplementary Material S2: Scans of Western blot films indicating the sizes of proteins covered.
