# Supplementary material for: Selective Inhibition of Histone Deacetylases 1/2/6 in Combination with Gemcitabine: A Promising Combination for Pancreatic Cancer Therapy
Source: Cancers (Basel). 2019 Sep 7;11(9):1327. doi: 10.3390/cancers11091327 (PMC6770665; doi:10.3390/cancers11091327)
Supplement: Supplementary file 1 [file cancers-11-01327-s001.pdf]

Supplemental Material S1. Densitometry analysis of Western blots (band intensity normalized to loading control)

Fig. 1A

DRUG		Panobinostat			Droxinostat			Mocetinostat			LMK-235		
Time (h)	0	4	8	24	4	24	0	4	8	24	4	8	24
Cleaved PARP	1.0	1.1	2.1	12.5	1.2	2.5	1.0	0.9	0.7	10.4	1.0	0.2	20.6
Cleaved Caspase 7	1.0	1.0	0.9	10.6	1.2	1.2	1.0	1.4	1.3	13.0	1.0	1.3	27.9

Fig. 1C

Time (h)	Control	LMK-235	Mocetinostat	Gemcitabine	Moce + LMK	Moce + LMK + Gem
Cleaved PARP	1.0	3.0	0.8	1.5	6.2	11.3

Fig. 2A

	MiaPaCa-2	Capan-1	BxPc-3	Panc-1	CFPAC
HDAC1	1.0	1.0	1.8	0.9	0.9
HDAC2	1.0	0.8	1.3	0.7	0.8
HDAC3	1.0	1.1	1.3	0.5	0.6
HDAC4	1.0	0.5	1.8	2.2	2.0
HDAC5	1.0	1.1	2.1	1.4	0.7
HDAC6	1.0	1.0	1.5	1.0	0.7

Fig. 2B

MiaPaCa-2							
siRNA	Non-Targeting	HDAC1	HDAC2	HDAC3	HDAC4	HDAC5	HDAC6
HDAC1	1.0	0.2	1.4	1.3	1.4	1.4	1.4
HDAC2	1.0	1.2	0.3	1.0	1.0	1.0	1.0
HDAC3	1.0	1.2	1.0	0.2	1.5	1.1	0.9
HDAC4	1.0	1.4	1.0	1.3	0.3	1.7	1.3
HDAC5	1.0	1.1	1.2	1.1	1.1	0.4	1.1
HDAC6	1.0	1.1	1.0	1.4	1.4	1.3	0.5

Capan-1							
siRNA	Non-Targeting	HDAC1	HDAC2	HDAC3	HDAC4	HDAC5	HDAC6
HDAC1	1.0	0.1	2.0	2.0	1.7	1.6	1.3
HDAC2	1.0	1.9	0.2	1.5	1.4	1.2	1.4
HDAC3	1.0	1.1	1.2	0.2	1.2	1.0	1.1
HDAC4	1.0	0.9	0.9	1.3	0.3	1.3	0.9
HDAC5	1.0	1.1	1.1	1.3	1.2	0.1	1.0
HDAC6	1.0	1.2	1.0	1.1	1.1	1.0	0.1

Fig. 2C

MiaPaCa-2						
siRNA	Non-Targeting	HDAC1	HDAC2	HDAC3	HDAC1/2	HDAC1/2/3
HDAC1	1.0	0.05	0.9	0.8	0.1	0.1
HDAC2	1.0	1.1	0.1	1.0	0.33	0.27
HDAC3	1.0	1.2	1.2	0.2	1.4	0.2

Capan-1			
siRNA	Non-Targeting	HDAC6	HDAC4/5/6
HDAC4	1.0	0.8	0.1
HDAC5	1.0	0.8	0.2
HDAC6	1.0	0.1	0.2

Capan-1			
siRNA	Non-Targeting	HDAC1/2	HDAC1/2/6
HDAC1	1.0	0.1	0.2
HDAC2	1.0	0.2	0.2
HDAC6	1.0	1.1	0.1

Fig. 4Bi

Capan-1					
ACY-1215 (μM)	0	0.5	1.0	5.0	10
ac-α-Tubulin	1.0	29.0	42.3	43.7	43.9
ac- Histone 3	0.0	0.0	0.0	0.5	1.0

MiaPaCa-2					
ACY-1215 (μM)	0	0.5	1.0	5.0	10
ac-α-Tubulin	1	10.4	25.2	26.0	24.3
ac- Histone 3	0.0	0.0	0.3	0.7	1.0

Fig. 5Ai

Capan-1				
	Control	Gem	Rom + ACY	Rom + ACY + Gem
Cleaved PARP	1.0	2.0	4.5	8.1
Cleaved Caspase 7	1.0	9.3	49.0	166.0

MiaPaca-2				
	Control	Gem	Rom + ACY	Rom + ACY + Gem
Cleaved PARP	1.0	2.0	15.0	27.1
Cleaved Caspase 7	1.0	2.5	48.7	88.0

Supplemental Material S2. Scans of Western blot films indicating the sizes of proteins covered

Fig. 1A

PARP

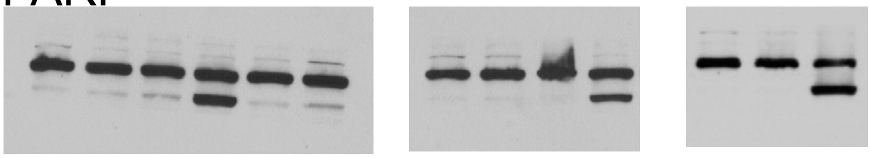

Blot was cut between 52 kDa and 72 kDa and between the 140 kDa and 260 kDa markers

Caspase 7

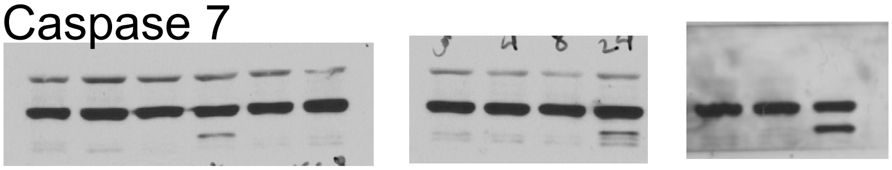

Blot was cut between 52 kDa and 42 kDa and at the 26 kDa marker

GAPDH

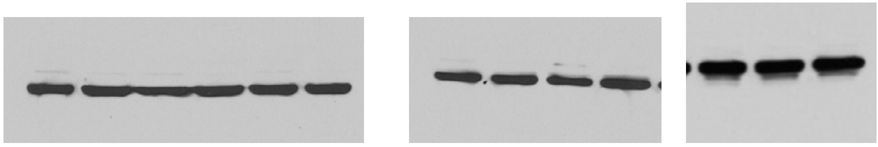

Blot was cut between 52 kDa and 42 kDa and between 35 kDa and 26 kDa markers

Fig. 1C

PARP

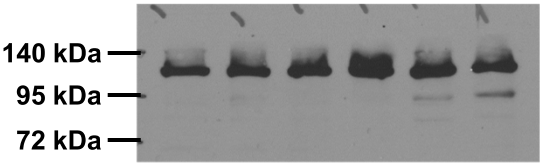

Blot was cut between 52 kDa and 72 kDa and between the 140 kDa and 260 kDa marker

GAPDH

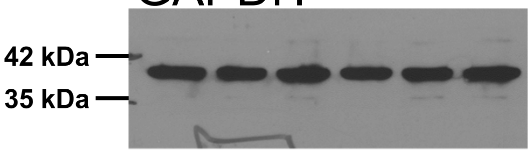

Blot was cut between 52 kDa and 42 kDa and between 35 kDa and 26 kDa markers

Fig. 2A

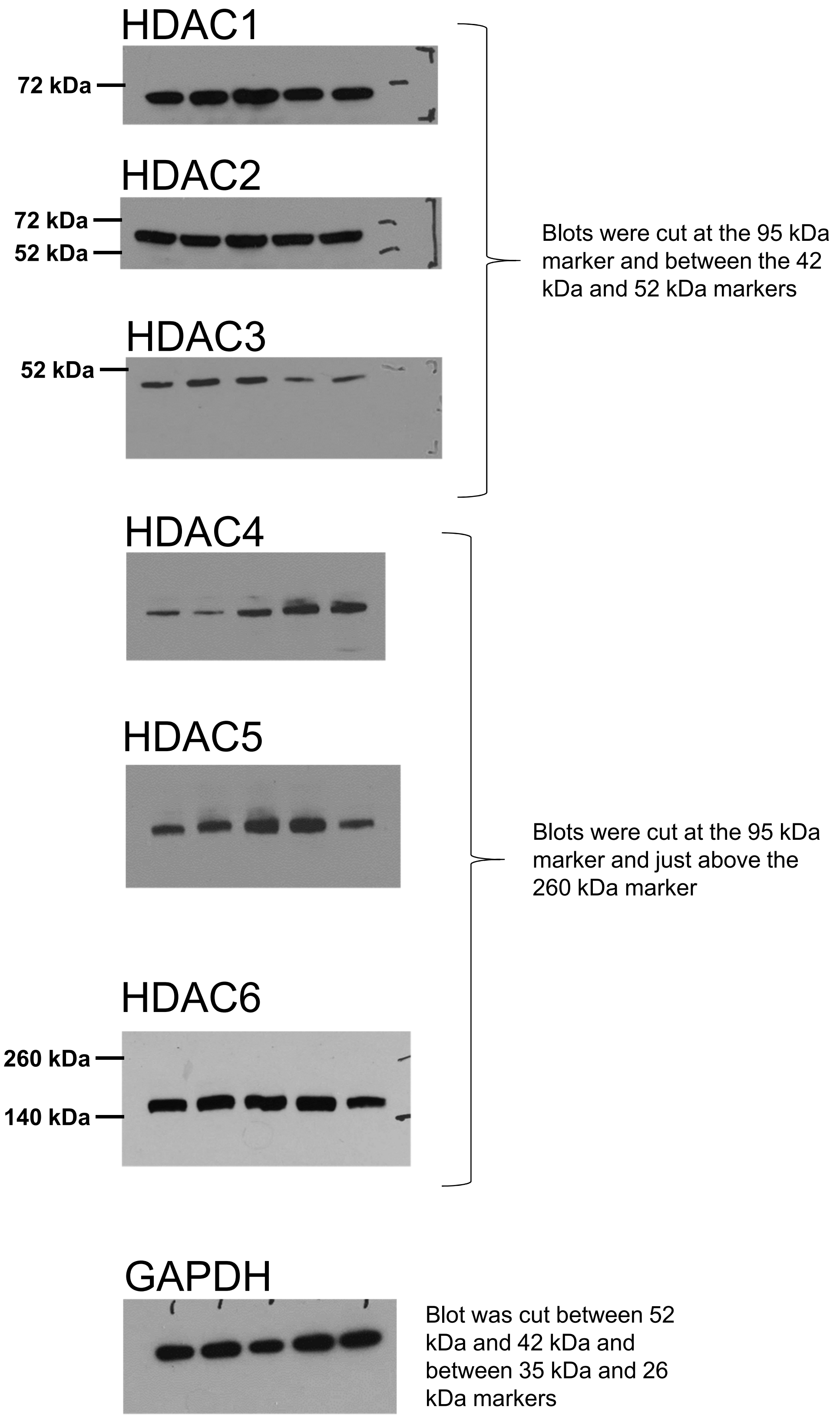
HDAC1
72 kDa
HDAC2
72 kDa
52 kDa
HDAC3
52 kDa
Blots were cut at the 95 kDa marker and between the 42 kDa and 52 kDa markers
HDAC4
HDAC5
HDAC6
260 kDa
140 kDa
Blots were cut at the 95 kDa marker and just above the 260 kDa marker
GAPDH
Blot was cut between 52 kDa and 42 kDa and between 35 kDa and 26 kDa markers

Fig. 2Bi

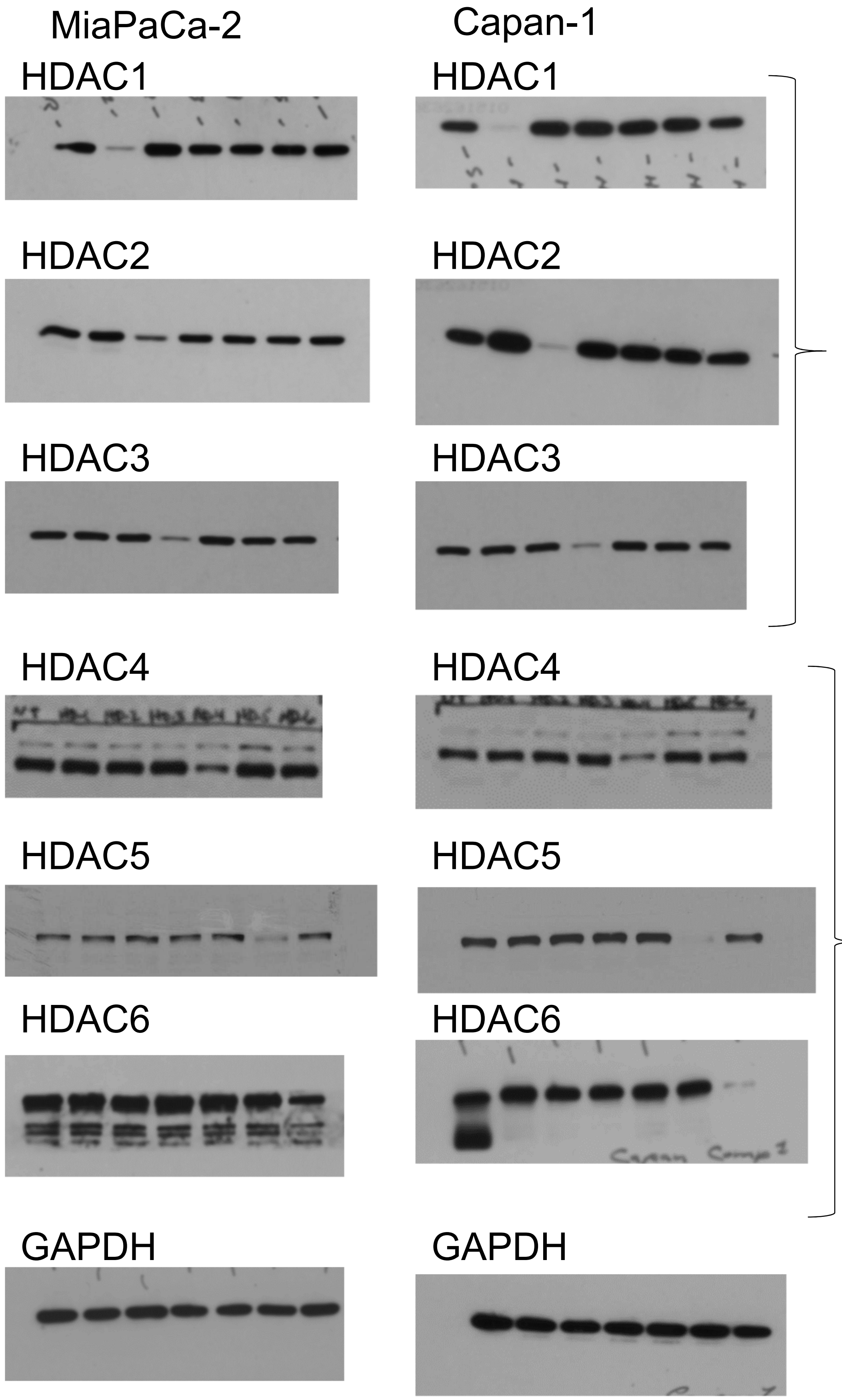

Blots were cut at the 95 kDa marker and between the 42 kDa and 52 kDa markers

Blots were cut at the 95 kDa marker and above the 260 kDa marker

Blot was cut between 52 kDa and 42 kDa and between 35 kDa and 26 kDa markers

Fig. 2Bii

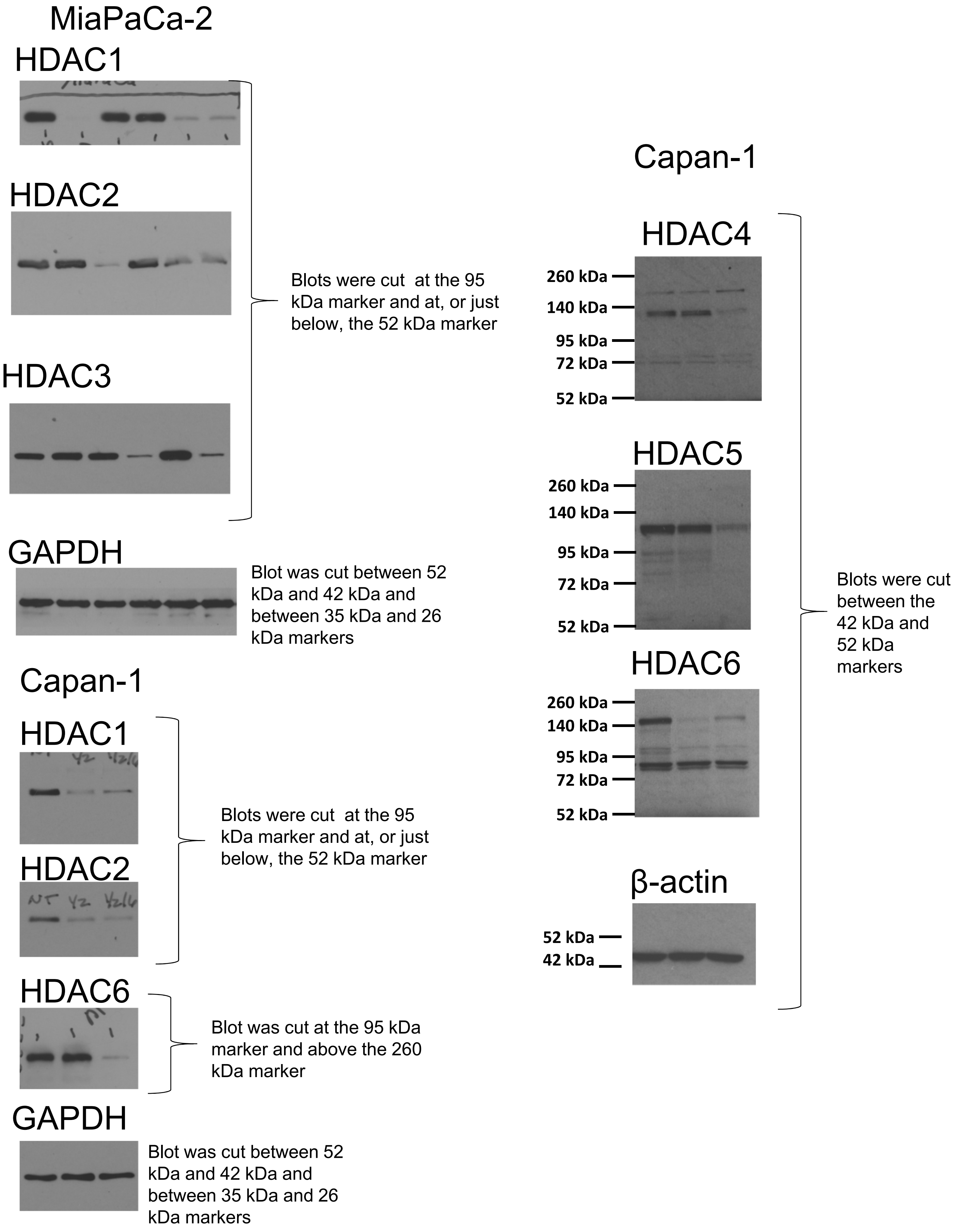
MiaPaCa-2
HDAC1
HDAC2
HDAC3
Blots were cut at the 95 kDa marker and at, or just below, the 52 kDa marker
GAPDH
Blot was cut between 52 kDa and 42 kDa and between 35 kDa and 26 kDa markers
Capan-1
HDAC1
HDAC2
Blots were cut at the 95 kDa marker and at, or just below, the 52 kDa marker
HDAC6
Blot was cut at the 95 kDa marker and above the 260 kDa marker
GAPDH
Blot was cut between 52 kDa and 42 kDa and between 35 kDa and 26 kDa markers
Capan-1
HDAC4
260 kDa
140 kDa
95 kDa
72 kDa
52 kDa
HDAC5
260 kDa
140 kDa
95 kDa
72 kDa
52 kDa
HDAC6
260 kDa
140 kDa
95 kDa
72 kDa
52 kDa
β-actin
52 kDa
42 kDa
Blots were cut between the 42 kDa and 52 kDa markers

Fig. 4B

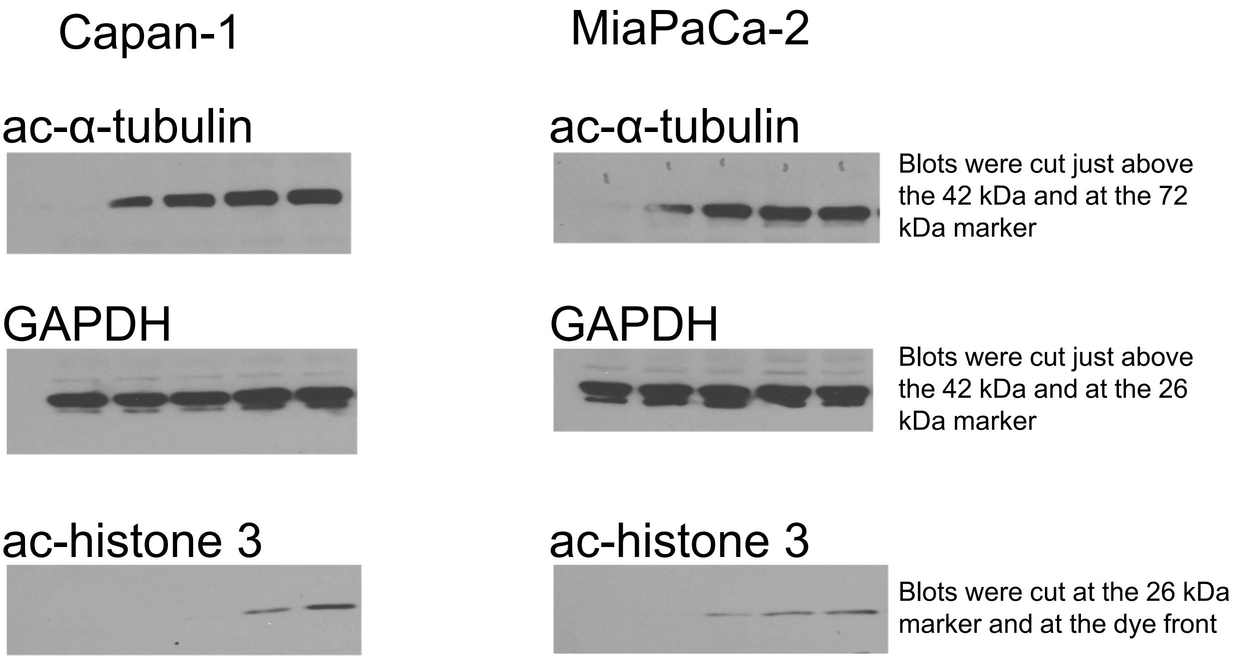
Capan-1
MiaPaCa-2
ac-α-tubulin
ac-α-tubulin
Blots were cut just above the 42 kDa and at the 72 kDa marker
GAPDH
GAPDH
Blots were cut just above the 42 kDa and at the 26 kDa marker
ac-histone 3
ac-histone 3
Blots were cut at the 26 kDa marker and at the dye front

Fig. 5A

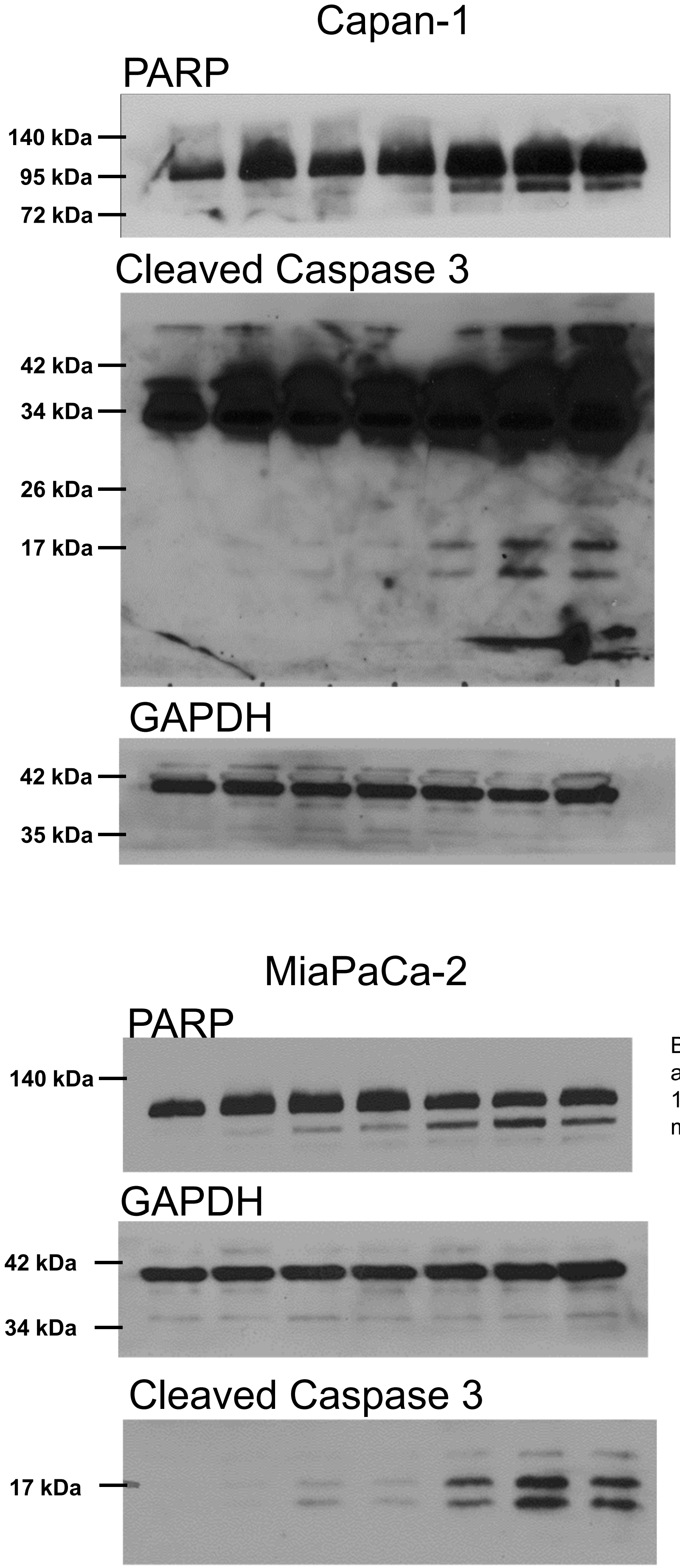

Blot was cut between 52 kDa and 72 kDa and between the 140 kDa and 260 kDa marker

Blot was cut at the 52 kDa marker

Blot was cut between 52 kDa and 42 kDa and between 35 kDa and 26 kDa markers

Blot was cut between 52 kDa and 72 kDa and between the 140 kDa and 260 kDa markers

Blot was cut between 52 kDa and 42 kDa and between 35 kDa and 26 kDa markers

Blot was cut between 35 kDa and 26 kDa and at the dye front
